# CNN-Based Smoker Classification and Detection in Smart City Application

**DOI:** 10.3390/s22030892

**Published:** 2022-01-24

**Authors:** Ali Khan, Somaiya Khan, Bilal Hassan, Zhonglong Zheng

**Affiliations:** 1 College of Mathematics and Computer Science, Zhejiang Normal University, Jinhua 321004, China; kaa503@zjnu.edu.cn; 2 School of Electronics Engineering, Beijing University of Posts and Telecommunications, Beijing 100876, China; somaiya.khan13@yahoo.com; 3 School of Automation Science and Electrical Engineering, Beihang University, Beijing 100191, China; bilz@live.com; 4 Key Laboratory of Intelligent Education Technology and Application of Zhejiang Province, Zhejiang Normal University, Jinhua 321004, China

**Keywords:** AI-based surveillance, smoker detection dataset, smoker classification, transfer learning

## Abstract

To better regulate smoking in no-smoking areas, we present a novel AI-based surveillance system for smart cities. In this paper, we intend to solve the issue of no-smoking area surveillance by introducing a framework for an AI-based smoker detection system for no-smoking areas in a smart city. Moreover, this research will provide a dataset for smoker detection problems in indoor and outdoor environments to help future research on this AI-based smoker detection system. The newly curated smoker detection image dataset consists of two classes, Smoking and NotSmoking. Further, to classify the Smoking and NotSmoking images, we have proposed a transfer learning-based solution using the pre-trained InceptionResNetV2 model. The performance of the proposed approach for predicting smokers and not-smokers was evaluated and compared with other CNN methods on different performance metrics. The proposed approach achieved an accuracy of 96.87% with 97.32% precision and 96.46% recall in predicting the Smoking and NotSmoking images on a challenging and diverse newly-created dataset. Although, we trained the proposed method on the image dataset, we believe the performance of the system will not be affected in real-time.

## 1. Introduction

The technological advancements in computing have led to networks of connected devices and sensors, which are central to the concept of smart cities. Governments all around the globe are embracing the idea of smart cities to improve the living standards of their people [1]. Adopting these technologies will enable cities to integrate the learning principles and requirements of smart city applications to create a smart environment [2,3] that is characterized by resilience, sustainability, improved quality of life, intelligent management and governance, etc.

The internet of things (IoT) has become an increasingly popular technology and has had a vital role in making smart city applications possible [4]. The IoT has played an important part in transforming various areas of life such as transportation [5], health [6], energy [7], education [8], surveillance [9], etc. Due to the IoT, where smart devices are connected to the internet, massive amounts of data are generated from devices such as computers, smartphones, cameras, sensors, etc. [10]. Artificial intelligence is useful for processing and analyzing these data, and it has taken the technological world to new heights. The field of computer vision has emerged because of the enormous amounts of data generated in IoT.

Deep learning for detection and recognition has become the backbone of computer vision technology [11]. The deep neural network (DNN), which uses the deep learning method [12], is categorized as a non-linear model and can learn the multi-level abstract representations of raw data. The traditional machine learning algorithms require prior feature extraction, whereas DNN handles this within the model. DNNs can handle large high-dimensional datasets such as images, video, voice or text. The convolutional neural network (CNN) is a category of feedforward DNNs used for the classification and clustering of images on the basis of similarity and detection of objects within a scene. The CNN is the reason for the tremendous growth in deep learning because it is powering significant advances in computer vision, which has many intriguing applications such as medical diagnosis, surveillance, self-driving cars, security, etc.

In recent years, CNNs have become an area of great interest among researchers. AlexNet [13], a deep convolutional neural network, was proposed as a strategy for faster training by using rectified linear units (ReLU) rather than sigmoid activation functions, which results in time efficiency in training. Moreover, to reduce overfitting, the model used data augmentation to generate more training samples and used a new regularization method known as a dropout. This became a focal point in neural networks, so much so that those techniques have become standard in training deep learning models. After the success of AlexNet, other CNN models such as VGG [14], Inception [15] and ResNet [16] etc. have also been proposed.

Smoking is a major global issue, which causes severe health crises and is a burden on the economy [17]. The World Health Organization (WHO), as of July 2021 [18], estimates that around 8 million people die every year because of smoking. Out of 8 million, 7 million deaths are due to direct smoking, while around 1 million deaths are due to passive smoking. The WHO also estimates that the world’s economy has to bear the burden of over USD 500 billion every year due to smoking. The WHO’s framework convention on smoking control lists the monitoring of smoking and prevention policies as measures that should be taken. Therefore, AI-based surveillance of no-smoking areas for detecting smokers as potential violators is vital. In this research, we propose a CNN-based solution for smoker detection in the no-smoking areas of a smart city. The main contributions of this work are:

1. Introduces the framework of the AI-based smoker detection system;
2. Provides a dataset for smoker detection problems in indoor and outdoor environments to help further work on this AI-based smoker detection system;
3. Proposes a transfer learning-based Inception-ResNet-V2 approach for the classification of smokers based on smoking and not-smoking people from a new image dataset;
4. Evaluates the proposed approach for in-depth analysis of the newly created smoker detection dataset for the smoker classification problem, and also compares it with other CNN models.

The paper is organized as follows: Section 2 details the related work. Section 3 explains the framework of the AI-based smoker detection system. Section 4 explains the proposed transfer learning-based Inception-ResNet-V2 approach for classification problem. Section 5 presents the details on the datasets. Section 6 details the performance evaluation of the proposed approach on the datasets and the comparative analysis with other CNN models, and the conclusion is given in Section 7.

## 2. Literature Review

In recent years, the development of surveillance systems has been phenomenal because of the development in camera lens technology and computer hardware technology. Since then, this domain has received much attention from researchers for integrating computer vision, deep learning and image processing into surveillance systems for automated detection, recognition and prediction of objects, and scenes based on the information gathered from the surveillance camera. Traditional surveillance methods, such as on-location patrolling or live CCTV, have limitations and are subject to human error as there can be a moment when the human eye cannot detect the object or scene, leading to major accidents or mishaps [19].

There have been many studies involving different applications of surveillance using computer vision technology [20]. These applications include crime prevention [21], human activity detection [22,23], traffic monitoring [24,25], pedestrian detection [26,27], jaywalker detection [28] vehicle identification and recognition [29], face recognition [30,31], motion detection [32], fire detection [33] and so on. These surveillance applications have led to research in various domains in recent years, focusing on computer vision-based solutions.

The smoker detection problem can be regarded as a human activity recognition problem. There have been numerous studies on solving the problem of human activity recognition through computer vision. In [34], the authors proposed a semi-supervised learning framework for human activity recognition where the distance-based reward rule is introduced as a labelling scheme based on the deep Q-network. The proposed method used long short-term memory (LSTM) for classification of the feature pattern extracted from motion data. Another method [35] was proposed for human activity recognition using channel state information (CSI). In the proposed method, CSI data are converted into images and these images are used for human activity classification using a 2D-CNN classifier.

There has been some research on smoking detection through different methods. Several studies focused on using hand gestures with a wrist inertial measurement unit (IMU) for smoking event detection. In [36], the authors proposed a smoking gesture detection method based on two 9-axis IMUs (accelerometer, magnetometer, and gyroscope). The elbow position relative to the wrist was the primary prediction metric for smoking. The random forest model used these features for smoking prediction. Tang et al. [37] proposed a machine learning method for puffing and smoking detection using data from a wrist accelerometer. Their proposed method consists of a two-layer model that integrates high-level smoking topography and low-level time domain features to detect puffing and then smoking.

Shoaib et al. [38] proposed a two-layer hierarchical smoking detection algorithm (HLSDA) for differentiating smoking behavior from different similar activities such as eating and drinking. A classifier is used in the first layer in the proposed method, followed by a context rule-based correction method that uses neighboring segments for accurate detection. Another method [39] for detecting smoking activity was proposed using IMU sensors in two levels, namely, puff and cigarette levels. The proposed method used support vector machine (SVM) and edge detection-based learning for feature detection of arm movements. In [40], the authors proposed a smoking detection method based on the regularity score of different hand-to-mouth gestures. The proposed method extracts the features from IMU sensors. Further, to quantify the regularity score, the authors proposed an unbiased autocorrelation approach for processing the temporal sequence of different hand gestures.

However, these studies have limited accuracy given the limited variations in the datasets that have been collected and the sensors’ ability to distinguish between similar body gestures. Few researchers have incorporated computer vision to solve this problem. To solve the problem of traditional supervision and the low precision of smoke alarms in an indoor environment, Rentao et al. [41] presented a deep learning-based solution based on YOLOv3-tiny, named Improved YOLOv3-tiny. Their proposed method inputs the images from their own created image dataset in an indoor environment. Another method proposed by Macalisang et al. [42] is based on transfer learning using YOLOv3 as the base model for detecting smokers. These two studies used YOLO, which promotes fast localization ability but lacks high accuracy of the considered problem. For the smoker classification problem, false alarms should be minimized for both classes. A large number of accurate detections in one class and a comparatively larger number of false alarms in another class can provide higher average precision and accuracy, but may not necessarily solve the desired problem. In [43], Zhang et al. presented a CNN model named SmokingNet based on the GoogleNet model for smoker detection. The proposed model inputs the images from the smoking video feed for detecting the smoker. Their research focused on accurate classification of smoking and not-smoking images with better consideration of the results of both the classes with performance metrics such as precision, recall and F1 score. Table 1 presents the comparative analysis of the computer vision-based smoker detection methods in the literature.

However, these CNN-based smoker detection methods through computer vision have limitations. The unavailability of the dataset or considering only cigarettes for the feature, for example, might affect the applicability of these solutions in other environments. Due to the limitations of the previous work in terms of dataset and prediction accuracy, this research focuses on developing a transfer learning-based solution for effective surveillance to ensure a healthy and smoke-free environment in a smart city. The main goal of our research is the accurate classification of Smoking and NotSmoking images with minimum false alarms, and at the same time, yielding better precision, recall, F1 score, average precision and AUC. The performance of our approach shows promising results in terms of prediction accuracy based on our newly curated dataset.

## 3. Smoker Detection System

**Figure 1:**
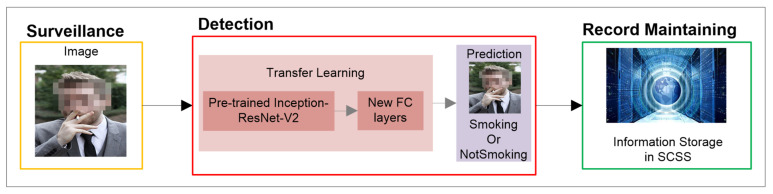
Smoker detection system model.

Smoker detection in no-smoking areas is inherently difficult due to the highly dynamic environment, varying atmospheric conditions, obstacles and seasonal changes. These factors highly influence the development stage of an automated algorithm for detecting smokers in real-time. Therefore, we propose a novel AI-based surveillance application to improve the environment in smart cities by detecting smokers in prohibited areas and implementing strict measures against them. This work focuses on the implementation of a CNN-based smoker detection system in prohibited areas. We consider that the mechanism for maintaining violator’s records in the SCSS is beyond the scope of the current work. The overview of the smoker detection system is illustrated in Figure 1.

**Figure 2:**
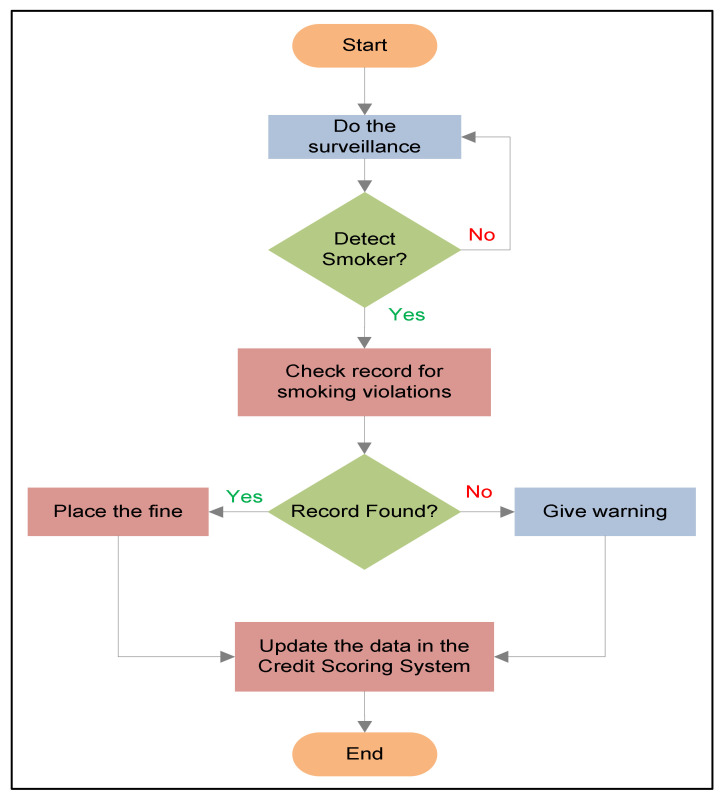
Flow diagram of working mechanism.

The continuous surveillance of no-smoking areas ensures strict regulation. The working mechanism of the smoker detection system is depicted in Figure 2. When a person standing or sitting in an indoor or outdoor public space takes out a cigarette and lights it, the continuous surveillance will help to detect the no-smoking violation through an automated detection system based on CNN. The detection system takes the images of the people as an input to the CNN model and outputs a prediction of whether the person is smoking or not, and then alerts the concerned department or people to ask the person to extinguish it. The images will be taken whenever the surveillance system detects a person in the no-smoking area to check for potential violators. This can be integrated with the social credit scoring system (SCSS), where previous violations related to smoking can be checked. If the person already has smoking violations, then a fine can be imposed on the smoker in a no-smoking area. On the contrary, if there is no previous record of smoking violations, a warning would be sent, and the database can be updated accordingly.

## 4. Proposed Approach

The success of CNNs in computer vision is due to the good results achieved by using large datasets such as ImageNet [44], MNIST [45], CIFAR [46], etc. Large datasets are critical for the performance of deep learning models. Neural networks are expected to perform better when using a large training dataset. Moreover, a comprehensive input space is also important as failure in testing may happen when the models are given new inputs. When working with supervised learning, one should know that CNN models show excellent performance in regard to generalization but are not good at extrapolating data for which they did not learn to extract representation.

**Figure 3:**
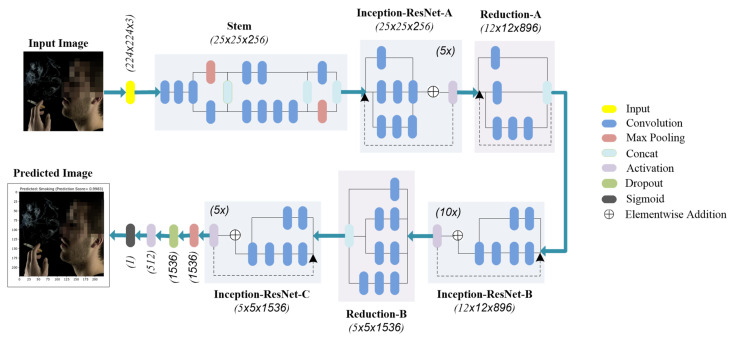
Inception-ResNet-V2 model for smoker classification.

By emphasizing the significance of the size of the dataset, real-world scenarios often face the problem of the availability of large datasets. Larger datasets take years to develop, and even a small dataset requires collaboration between different disciplines to acquire data. Transfer learning offers a promising solution for better results with a limited dataset to overcome dataset limitations. Figure 3 illustrates the Inception-ResNet-V2 model used in this study to evaluate the performance of the smoker classification task on a smoker detection dataset.

Transfer learning, a deep learning method, uses a pre-trained model to learn a completely new task with a different dataset. Lately, the transfer learning approach has been used extensively in solving problems with limited available datasets [47], thus facilitating unique applications in various fields. Following this technique, our research utilized the Inception-ResNet-V2 model pre-trained on the ImageNet dataset. We re-trained the model by modifying the fully connected layers for transfer learning.

### 4.1. Inception-ResNet-V2 Model

The Inception-ResNet-V2 model [48] is a DNN that is trained on the ImageNet database. It is 164 layers deep and is a hybrid model, which has significantly improved its performance in terms of recognition tasks compared to its predecessors [49]. The model is based on combining the Inception structure with the residual connections. The successive Inception-ResNet blocks in the model comprise many different convolution layers combined with residual connections, and provide useful features extraction. The residual connections help circumvent the degradation problem caused by the deep structure and reduce the training time.

We used the Inception-ResNet-V2 model by freezing the weights and leveraging the pre-trained convolutional base. Further, we employed our own fully connected and classification layers to distinguish between the two classes using the transfer learning methodology. The starting layers of the neural network learn the generic features, whereas the last layers are for more specific features of the problem. So, we added our layer with the ReLu activation function. As the problem considered in this research is binary, the classifier has a single output of 0 or 1, in our case, NotSmoking or Smoking. The original classifier in the Inception-ResNet-V2 model consisted of 1000 neurons as it was designed to distinguish between 1000 different objects using the ImageNet dataset.

### 4.2. Activation Functions

The weights of the Inception-ResNet-V2 model were frozen, and new fully connected dense layers were added. An activation function is important for the process of optimization. The rectified linear unit (ReLU) and sigmoid activation functions were used for the proposed method. The ReLU activation function acts as a linear function and learns complex features of the data. As this work focuses on smoker detection, for probability prediction as the output, the sigmoid function (also called logistic function) was used as it is differentiable, and it has a signal output between 0 and 1. The ReLU and sigmoid functions are given as follows:

(1) $$ReLU\;\left(x\right)=max\left(0,\;x\right)$$

(2) $$Sigmoid\;\left(x\right)=\frac{1}{\left(1+e^{-x}\right)}$$

### 4.3. Optimization Function

For training the proposed transfer learning-based Inception-ResNet-V2, stochastic gradient descent (SGD) was used as an optimizer. SGD is the most common optimization algorithm for training neural networks. It is a first-order optimization method. The SGD algorithm updates the parameters per iteration, and the update of each parameter is computed based on a few training samples. This reduces variance in the parameter update and provides stable convergence. The SGD optimizer equation is given as:

(3) $$\theta =\theta -\eta \Delta_{\theta }J\left(\theta ;x^{\left(i\right)};y^{\left(i\right)}\right)$$

where θ is the model parameters, $\Delta_{\theta }J\left(\theta \right)$ is the gradient of the loss function with regard to parameter θ and $x^{\left(i\right)}$, $y^{\left(i\right)}$ are the training examples.

## 5. Dataset

We have considered a binary classification problem in this study, so we collected and arranged the images related to the classification problem of Smoking and NotSmoking to facilitate research on new methodologies for the smoker detection domain. We acquired the images from various online sources and with multiple angles and views for better training the model to discriminate the smokers from non-smokers. In our dataset, the Smoking class contains images of a person smoking a cigarette with visible smoke or a lighted cigarette in their mouth. In contrast, the NotSmoking class contains images of a person not smoking but who has slightly similar body actions where the hand gestures and body index are almost the same, such as drinking water, coughing, taking an inhaler, etc. Our dataset consists of 1120 images, where 560 images belong to the Smoking class and the remaining 560 belong to the NotSmoking class.

### 5.1. Dataset Preprocessing and Partitioning

**Figure 4:**
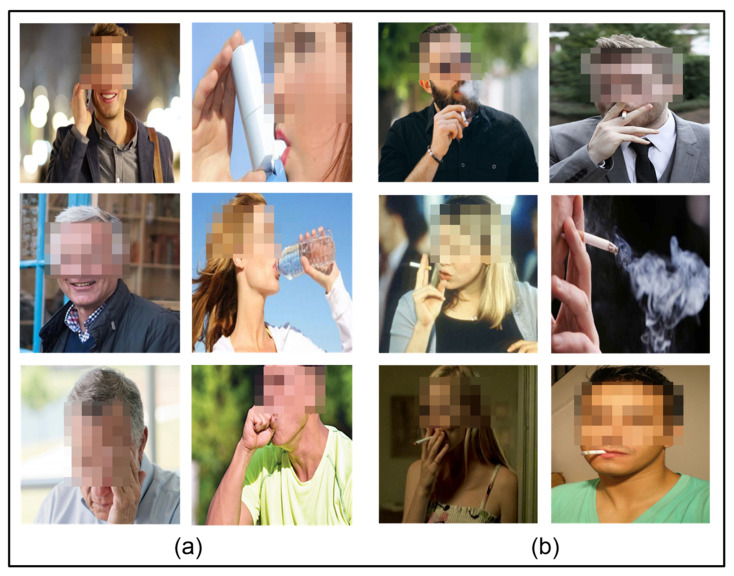
Images of (**a**) NotSmoking class and (**b**) Smoking class.

After curating the dataset, it needs to undergo some cleaning processes to show a clear representation of the considered problem. The dataset was preprocessed using different methods such as cropping, resizing, etc. The images in the curated dataset have unwanted backgrounds given the considered problem. Therefore, the images were cropped to filter out the unwanted backgrounds and obtain only the desired part of the problem. After cropping, the images in the dataset were resized to a common resolution of 250×250. Figure 4 shows some representative images from the dataset. After this preprocessing, the dataset was partitioned for training and testing purposes. We considered 80% of the data for training and validation purposes and 20% for the testing. Further, 80% of the data was split into training and validation data, 716 images for training and 180 for validation. The testing data consisted of 224 images.

### 5.2. Data Augmentation

**Figure 5:**
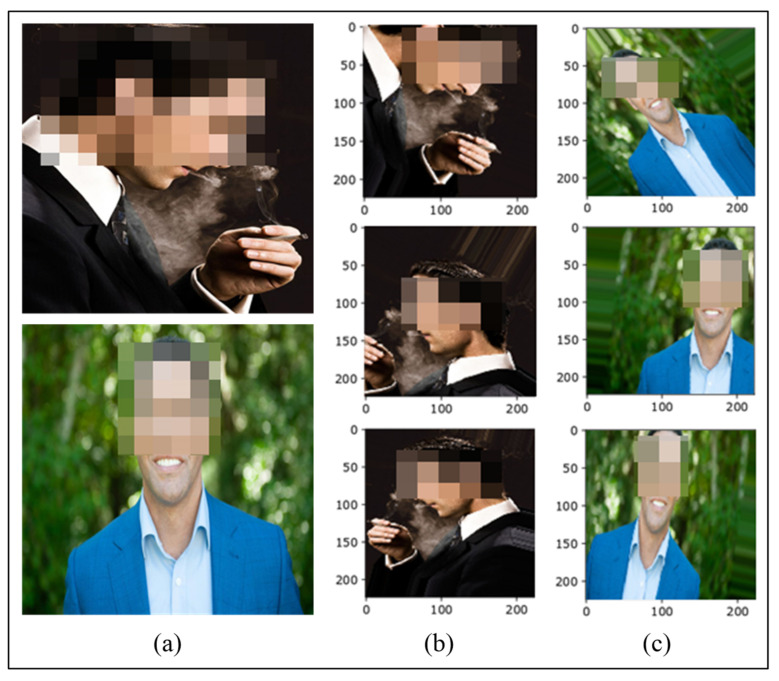
Images of (**a**) resized, random augmentations on (**b**) Smoking and (**c**) NotSmoking.

Although using deep learning models substantially improve the results, higher detection accuracy requires a large training dataset. Otherwise, the model is prone to the issue of over-fitting due to dataset limitations, whereby the trained model does not show good generalization and cannot perform well on new and unseen data. Therefore, we adopted a data augmentation strategy on the training dataset to overcome this issue in this research. We performed various augmentations such as resizing, scaling, flipping, shifting, etc. as illustrated in Figure 5. Firstly, images in the dataset were resized to a common resolution of 224×224, which is also the accepted input of the Inception-ResNet-V2 model. Afterwards, random augmentations were performed, including scaling the image up to a factor of 0.2, rotation of the image up to a 50° angle, horizontal or vertical translation by a factor of 0.2. We also applied shear-based transformation up to a factor of 0.2.

## 6. Performance Evaluation

In our research, the smoker dataset was classified, and results were analyzed for a transfer learning-based solution using Inception-ResNet-V2 on the smoker detection dataset, and we compared the performance with other CNN models. The simulations were done in Python 3.7 using Tensorflow/Keras libraries. The system configurations for the simulations were Dell i9-11950H, 64GB DDR4, 4GB NVIDIA T600. The details of the performance analysis are presented in the subsequent subsections.

### 6.1. Simulation Parameters Selection

We performed empirical testing to select optimal values against each hyper-parameter. The input image size for the simulation was set to be 224×224. After comprehensive testing with different values for the batch size and learning rate, the parameters selected for training purposes in our simulation were specified as shown in Table 2.

### 6.2. Performance Metrics

The proposed transfer learning-based Inception-ResNet-V2 model was evaluated for accurate classification of smoking and not-smoking images in the smoker detection dataset. Moreover, it was compared with other CNN models on various performance metrics such as prediction accuracy, sensitivity (Recall), specificity, error rate, positive predictive values (Precision), negative predictive values ($PV_{n}$), false negative rate (FNR), false positive rate (FPR), false discovery rate (FDR), and F1 score. The equations for the performance metrics are given by:

(4) $$Prediction\;Accuracy=\frac{T_{p}+T_{n}}{T_{p}+T_{n}+F_{p}+F_{n}}$$

(5) $$Precision\;\left(PV_{p}\right)=\frac{T_{p}}{T_{p}+F_{p}}$$

(6) $$Recall\;\left(Sensitivity\right)=\frac{T_{p}}{T_{p}+F_{n}}$$

(7) $$Specificity=\frac{T_{n}}{T_{n}+F_{p}}$$

(8) $$PV_{n}=\frac{T_{n}}{T_{n}+F_{n}}$$

(9) FPR=1−Specificity

(10) FNR=1−Sensitivity

(11) $$FDR=1-PV_{p}$$

(12) $$F1\;score=2*\left(\frac{Precision*Recall}{Precision+Recall}\right)$$

(13) $$E_{r}=\frac{F_{p}+F_{n}}{T_{p}+T_{n}+F_{p}+F_{n}}$$

$T_{n}$ and $T_{p}$ are true negatives (accurately identified as NotSmoking) and true positives (accurately identified as Smoking), respectively. False positives $F_{p}$ are those NotSmoking images labelled as Smoking, while false negatives $F_{n}$ are those Smoking images that are classified as NotSmoking. Precision represents the ratio of correct positive results and positive results predicted by the classifier. The Recall is the ratio of correct positive results and all relevant samples that should have been predicted as positive. Specificity, also called the true negative rate, is the ratio of correct negative results and negative results predicted by the classifier. $PV_{n}$ is the negative predictive value. FDR represents the false discovery rate whereas FPR and FNR represent the false positive and false negative rates, respectively. $E_{r}$ is the error rate, which is the incorrect predictions for the total test samples. The F1 score is the harmonic mean between precision and recall, which shows how the classifier predicts correctly.

### 6.3. Image Processing of the Proposed Approach

In our proposed method, the whole image with an input size of 224×224 was fed to the neural network. The neural network extracted the features based on the smoke and cigarette along with the hand gesture on the mouth. This can be noted from the results as well. The false classifications indicated that the images with a similar hand gesture and a background of similar color to the cigarette with no smoke were misclassified by the proposed method. At the time, the authors considered images with only one person. Having more than one person in the image will not affect the processing steps as the input is the image size, which in our proposed method is 224×224. Moreover, it will not degrade the performance as there would be more patches of smoke and cigarette in the image for feature extraction.

### 6.4. Performance Analysis

This subsection presents a detailed performance analysis of the proposed transfer learning-based Inception-ResNet-V2 approach based on the smoker detection dataset. Subsequently, a comparative analysis of the performance of Xception [50], Inception [49], NASNetMobile [51] and VGG19 [14] models was made based on the smoker detection dataset.

#### 6.4.1. Performance of Proposed Approach on the Smoker Detection Dataset

According to the results, the proposed method shows 0.9687 accuracy with a 0.0312 error rate, 0.0357 FPR, 0.0354 FNR and 0.0268 FDR, which are good results considering the very new and diverse smoker detection dataset for smoker classification. The performance of the rest of the evaluation metrics on the smoker detection dataset is presented as follows.

##### Confusion Matrix

**Figure 6:**
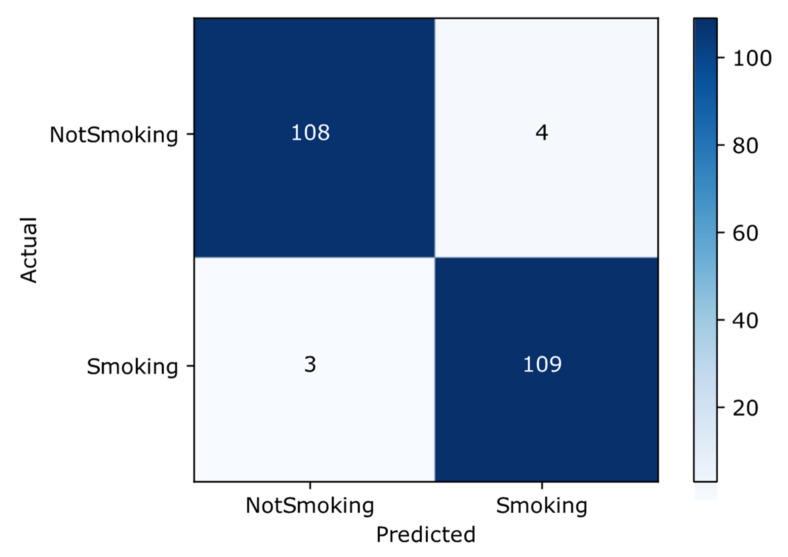
Confusion matrix.

The confusion matrix is a tool that provides a predictive analysis of the classification task. Accuracy alone can sometimes be misleading; however, a confusion matrix can provide a better idea of the model in regard to what it is classifying correctly and the errors it is making. Figure 6 shows the confusion matrix of the proposed transfer learning-based Inception-ResNet-V2 approach. By looking at the larger diagonal values and small values off the diagonal of the confusion matrix, we can deduce that the proposed approach shows promising results for the smoker classification problem. It has 109 and 108 true positive and true negative results, respectively, whereas the false positive and negative results are 4 and 3, respectively.

##### ROC Curve

**Figure 7:**
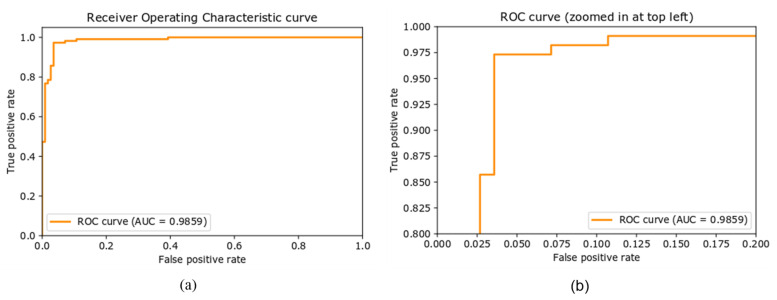
(**a**) Receiver operative characteristic curve (ROC), (**b**) ROC curve with zoomed representation.

The receiver operative characteristic (ROC) curve is another performance metric for the classification task at different settings of the threshold. ROC curve is plotted with the true positive rate, also called recall or sensitivity, against the false positive rate (FPR). The area under the curve (AUC) represents the degree or measure of separability of the classes, that is, it reveals how well the model can distinguish between the classes. The higher the value of AUC, the better the performance of the model in predicting the classes correctly. Figure 7 shows the ROC curve of the proposed approach on the newly created dataset. The AUC value of 0.9855 means that there is a 98.55% chance that the model will distinguish correctly between positive and negative classes.

##### Precision–Recall Curve

**Figure 8:**
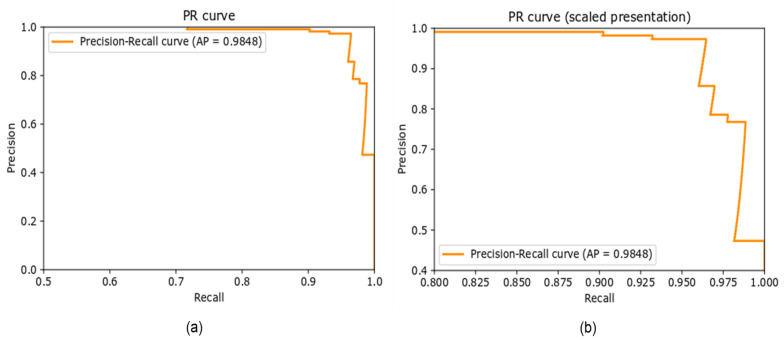
(**a**) Precision–recall curve, (**b**) scaled representation of Precision-recall curve.

The precision–recall curve (PR curve) is a graphical representation of the recall on the x-axis and precision on the y-axis. The closer the curve is to the upper right corner, the better the performance of the model in terms of prediction. Figure 8 illustrates the PR curve for the proposed approach on the newly created dataset. The AP is 0.9848 for the classification of the smoker detection dataset evaluated in the proposed approach.

##### True Predictions

**Figure 9:**
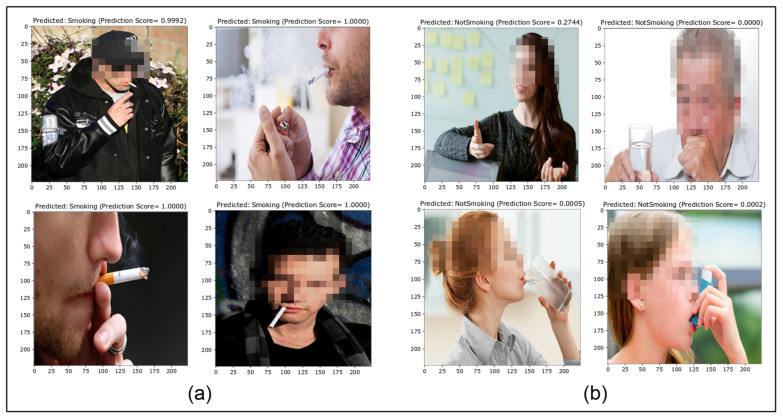
True prediction (**a**) positives and (**b**) negatives.

This presents the images correctly predicted for both positive and negative classes. Figure 9 shows some of the true positive and negative classifications. The proposed approach performed better for the newly-created smoker detection dataset with 217 true predictions out of 224.

##### False Negatives

**Figure 10:**
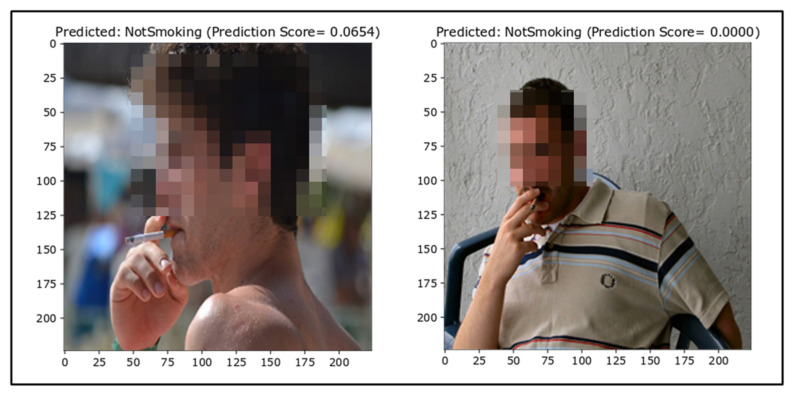
False negatives (ground truth = Smoking).

For smoker detection, the number of false negatives should be very minimal. False negatives are depicted in Figure 10. The false negative results show that images with a background (i.e., a crowd or other objects) are falsely classified. The false classification of Smoking images as NotSmoking is due to the spatial resolution, which plays a vital role in computer vision. Images with better and clearer resolution make it easier for the model to generalize better. During the selection of hyperparameters, it was noticed that changing the image size significantly affected the accuracy.

Moreover, there were some images where the background was out of focus, resulting in confusion for the neural network and the inability to differentiate between the cigarette and background pixels. Another reason for the falsely classified Smoking images might be due to the lack of a considerable number of varying images in the dataset. Neural networks are poor at generalizing situations for which they are not trained, so this might be another reason as some images in the test dataset were new to the model, that is, they lacked representation of like images in the training data.

##### False Positives

**Figure 11:**
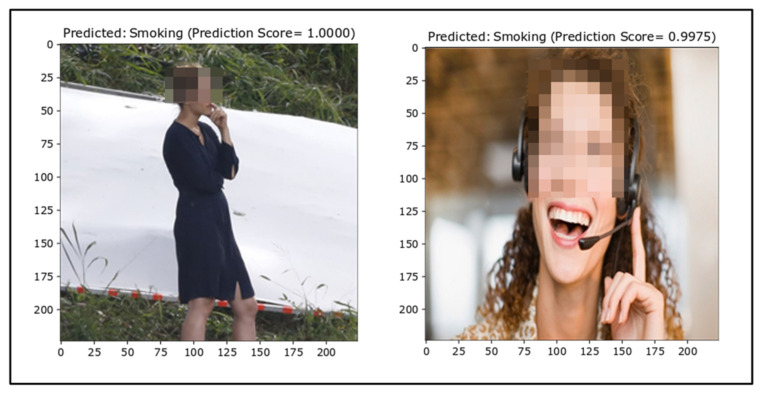
False positives (ground truth = NotSmoking).

Subsequently, the same issue was noticed for false positive outcomes. The percentage of false alarms is vital for smoker detection as it impacts on the reliability and applicability of the classifier in real-life applications. The false positive images are depicted in Figure 11. Some of the NotSmoking images might have been labeled as Smoking because of the diversity in the dataset and a lack of similar images in the training dataset.

#### 6.4.2. Comparison with Other Models

We have evaluated the smoker detection dataset on different CNN models such as InceptionV3 [49], Xception [50], NASNetMobile [51], VGG19 [14]. Table 3 shows that the proposed transfer learning-based Inception-ResNet-V2 has better performance in terms of prediction accuracy, precision, recall, AUC and AP in regard to our newly created smoker detection dataset. The InceptionV3 showed better results for the smoker detection dataset than the other models, followed by Xception. VGG19 performed worse as compared to the other models but still has considerable accuracy, precision, AUC and AP for a unique problem with a new and diverse dataset.

#### 6.4.3. Comparison with Other Smoker Classification Approaches

The metrics considered by the authors for SmokingNet [43] were prediction accuracy, precision, recall, F1 score and AUC. Their proposed method was applied to the local dataset and showed an accuracy of 0.90 with 0.90 precision and recall while AUC is 0.95. In contrast to this, our proposed approach on the smoker detection dataset showed a 0.9687 accuracy. Table 4 shows the comparative analysis of the proposed approach on the smoker detection dataset with SmokingNet. As depicted in Table 3, the accuracy of the proposed approach on our dataset shows better results compared to the other smoker classification approach, SmokingNet.

## 7. Conclusions

In this research work, to better regulate the ban on smoking in outdoor no-smoking areas, we presented a novel idea for an AI-based surveillance system for smart cities. We intended to solve the issue of no-smoking area surveillance by introducing a framework for an AI-based detection system of smokers in no-smoking areas. Moreover, this research has provided a dataset for the smoker detection problem in indoor and outdoor environments to help future research on this AI-based smoker detection system. The newly curated smoker detection image dataset consists of two classes, Smoking and NotSmoking. Further, to classify the Smoking and NotSmoking images, we proposed a transfer learning-based solution using the pre-trained InceptionResNetV2 model. The performance of the proposed approach for predicting smokers and not-smokers has been evaluated and compared with other CNN methods using different performance metrics. The proposed transfer learning-based InceptionResNetV2 achieved an accuracy of 96.87% with 97.32% precision and 96.46% recall in predicting the Smoking and NotSmoking images using a challenging and diverse dataset. Although, we trained the proposed method on an image dataset, we believe the performance of the system will not be affected in real-time.

## Figures and Tables

**Table 1:** Comparative analysis of the computer vision-based smoker detection methods.

Proposed Methods	Dataset Type	Dataset Availability	Tasks	Accuracy	Precision	Recall	AUC	F1 score	AP
Improved YOLOv3-tiny [40]	Images	Not open sourced	Localization	-	-	-	-	-	0.85
YOLOv3 [41]	Images	Not open sourced	Localization	0.90	-	-	-	-	-
SmokingNet [42]	Images	Not open sourced	Classification	0.90	0.90	0.90	0.95	0.90	-

**Table 2:** Simulation parameters.

Parameters	Value
Input size	224×224
Optimizer	Stochastic gradient descent
Loss	Binary cross-entropy
Learning rate	0.01
Batch Size	64
Epochs	50
Steps per epochs	100
Validation steps	100

**Table 3:** Performance analysis.

Metrics	Proposed Approach	Xception [50]	InceptionV3 [49]	NASNetMobile [51]	VGG19 [14]
$T_{p}$	109	98	105	95	91
$T_{n}$	108	104	104	103	96
$F_{p}$	4	8	8	9	16
$F_{n}$	3	14	7	17	21
Precision	0.9732	0.9245	0.9292	0.9135	0.8505
Recall	0.9646	0.8750	0.9375	0.8482	0.8125
$PV_{n}$	0.9729	0.8813	0.9369	0.8583	0.8205
Specificity	0.9643	0.9286	0.9286	0.9196	0.8571
FPR	0.0357	0.0714	0.0714	0.0804	0.1429
FNR	0.0354	0.1250	0.0625	0.1518	0.1875
FDR	0.0268	0.0755	0.0708	0.0865	0.4950
F1 score	0.9688	0.8990	0.9333	0.8796	0.8311
Prediction Accuracy	0.9687	0.9018	0.9330	0.8839	0.8348
$E_{r}$	0.0312	0.0982	0.0669	0.1161	0.1651
AUC	0.9859	0.9675	0.9776	0.9558	0.9208
AP	0.9848	0.9656	0.9762	0.9494	0.9221

**Table 4:** Comparative analysis with another smoker classification approach.

Metrics	SmokingNet [42]	Proposed Approach
Prediction Accuracy	0.90	0.9687
Precision	0.90	0.9732
Recall	0.90	0.9646
F1 score	0.90	0.9688
AUC	0.95	0.9859

## Data Availability

The dataset associated with the findings of this research work will be available upon request.

## References

[1] Jimenez C.E., Solanas A., Falcone F., Jimenez-Gomez C.E. (2014). E-Government Interoperability: Linking Open and Smart Government. Computer.

[2] Duan L., Lou Y., Wang S., Gao W., Rui Y. (2018). AI-Oriented Large-Scale Video Management for Smart City: Technologies, Standards, and Beyond. IEEE MultiMedia.

[3] Shao Z., Cai J., Wang Z. (2017). Smart Monitoring Cameras Driven Intelligent Processing to Big Surveillance Video Data. IEEE Trans. Big Data.

[4] Kim T., Ramos C., Mohammed S. (2017). Smart city and IoT. Future Gener. Comput. Syst..

[5] Brincat A.A., Pacifici F., Martinaglia S., Mazzola F. The Internet of Things for Intelligent Transportation Systems in Real Smart Cities Scenarios. Proceedings of the 2019 IEEE 5th World Forum on Internet of Things (WF-IoT).

[6] Yang P., Stankevicius D., Marozas V., Deng Z., Liu E., Lukosevivius A., Dong F., Xu L., Min G. (2018). Life logging data validation model for internet of things enabled personalized healthcare. IEEE Trans. Syst. Man Cybern. Syst..

[7] Bedi G., Venayagamoorthy G.K., Singh R. (2020). Development of an IoT-Driven Building Environment for Prediction of Electric Energy Consumption. IEEE Internet Things J..

[8] Mircea M., Stoica M., Ghilic-Micu B. (2021). Investigating the Impact of the Internet of Things in Higher Education Environment. IEEE Access.

[9] Rego A., Canovas A., Jimenez J.M., Lloret J. (2018). An Intelligent System for Video Surveillance in IoT Environments. IEEE Access.

[10] Hsiao Y.C., Wu M.H., Li S.C. (2021). Elevated Performance of the Smart City—A Case Study of the IoT by Innovation Mode. IEEE Trans. Eng. Manag..

[11] Khan A.I., Al-Habsi S. (2020). Machine Learning in Computer Vision. Procedia Comput. Sci..

[12] LeCun Y., Bengio Y., Hinton G. (2015). Deep learning. Nature.

[13] Krizhevsky A., Sutskever I., Hinton G.E. (2017). Imagenet classification with deep convolutional neural networks. Commun. ACM.

[14] Simonyan K., Zisserman A. Very deep convolutional networks for large-scale image recognition. Proceedings of the 3rd International Conference on Learning Representations (ICLR).

[15] Szegedy C., Liu W., Jia Y., Sermanet P., Reed S., Anguelov D., Erhan D., Vanhoucke V., Rabinovich A. Going deeper with convolutions. Proceedings of the IEEE Conference on Computer Vision and Pattern Recognition (CVPR).

[16] He K., Zhang X., Ren S., Sun J. Deep Residual Learning for Image Recognition. Proceedings of the IEEE Conference on Computer Vision and Pattern Recognition (CVPR).

[17] Ekpu V.U., Brown A.K. (2015). The Economic Impact of Smoking and of Reducing Smoking Prevalence: Review of Evidence. Tob. Use Insights.

[18] World Health Organization Tobacco Fact Sheet. https://www.who.int/news-room/fact-sheets/detail/tobacco.

[19] La Vigne N.G., Lowry S.S., Markman J.A., Dwyer A.M. (2011). Evaluating the Use of Public Surveillance Cameras for Crime Control and Prevention: A Summary.

[20] Tsakanikas V., Dagiuklas T. (2018). Video surveillance systems-current status and future trends. Comput. Electr. Eng..

[21] Moon H.-M., Chae S.-H., Moon D., Chung Y., Pan S.B. (2011). Intelligent video surveillance system using two-factor human information. Telecommun. Syst..

[22] Lin L., Wang K., Zuo W., Wang M., Luo J., Zhang L. (2015). A Deep Structured Model with Radius–Margin Bound for 3D Human Activity Recognition. Int. J. Comput. Vis..

[23] Cao S., Nevatia R. Exploring deep learning based solutions in fine grained activity recognition in the wild. Proceedings of the 2016 23rd International Conference on Pattern Recognition (ICPR).

[24] Ma C., Liu D., Peng X., Li L., Wu F. (2019). Traffic surveillance video coding with libraries of vehicles and background. J. Vis. Commun. Image Represent..

[25] Vishnu V.C.M., Rajalakshmi M., Nedunchezhian R. (2017). Intelligent traffic video surveillance and accident detection system with dynamic traffic signal control. Clust. Comput..

[26] Zhang S., Cheng D., Gong Y., Shi D., Qiu X., Xia Y., Zhang Y. (2018). Pedestrian search in surveillance videos by learning discriminative deep features. Neurocomputing.

[27] Lee J.H., Seo C.J. (2019). Deep Learning based Pedestrian Detection and Tracking System using Unmanned Aerial Vehicle and Prediction Method. Int. J. Innov. Technol. Explor. Eng..

[28] Khan S., Teng Y., Cui J. Pedestrian Traffic Lights Classification Using Transfer Learning in Smart City Application. Proceedings of the 13th International Conference on Communication Software and Networks (ICCSN).

[29] Dong H., Wang X., Zhang C., He R., Jia L., Qin Y. (2018). Improved Robust Vehicle Detection and Identification Based on Single Magnetic Sensor. IEEE Access.

[30] Yang S., Luo P., Loy C.-C., Tang X. From Facial Parts Responses to Face Detection: A Deep Learning Approach. Proceedings of the IEEE International Conference on Computer Vision (ICCV).

[31] Rao Y., Lu J., Zhou J. Attention-Aware Deep Reinforcement Learning for Video Face Recognition. Proceedings of the IEEE International Conference on Computer Vision (ICCV).

[32] Conte D., Foggia P., Percannella G., Tufano F., Vento M. (2010). A Method for Counting Moving People in Video Surveillance Videos. EURASIP J. Adv. Signal Process..

[33] Muhammad K., Ahmad J., Baik S.W. (2018). Early fire detection using convolutional neural networks during surveillance for effective disaster management. Neurocomputing.

[34] Zhou X., Liang W., Kevin I., Wang K., Wang H., Yang L.T., Jin Q. (2020). Deep-Learning-Enhanced Human Activity Recognition for Internet of Healthcare Things. IEEE Internet Things J..

[35] Moshiri P.F., Shahbazian R., Nabati M., Ghorashi S.A. (2021). A CSI-Based Human Activity Recognition Using Deep Learning. Sensors.

[36] Parate A., Chiu M.C., Chadowitz C., Ganesan D., Kalogerakis E. Risq: Recognizing smoking gestures with inertial sensors on a wristband. Proceedings of the 12th Annual International Conference on Mobile Systems.

[37] Tang Q., Vidrine D.J., Crowder E., Intille S.S. Automated detection of puffing and smoking with wrist accelerometers. Proceedings of the 8th International Conference on Pervasive Computing Technologies for Healthcare.

[38] Shoaib M., Scholten H., Havinga P.J., Incel O.D. A hierarchical lazy smoking detection algorithm using smart watch sensors. Proceedings of the IEEE 18th International Conference on e-Health Networking, Applications and Services (Healthcom).

[39] Raiff B.R., Karatas C., McClure E.A., Pompili D., Walls T.A. (2014). Laboratory validation of inertial body sensors to detect cigarette smoking arm movements. Electronics.

[40] Senyurek V.Y., Imtiaz M.H., Belsare P., Tiffany S., Sazonov E. (2019). Smoking detection based on regularity analysis of hand to mouth gestures. Biomed. Signal Process. Control..

[41] Rentao Z., Mengyi W., Zilong Z., Ping L., Qingyu Z. Indoor Smoking Behavior Detection Based on YOLOv3-tiny. Proceedings of the 2019 Chinese Automation Congress (CAC).

[42] Macalisang J.R., Merencilla N.E., Ligayo M.A.D., Melegrito M.P., Tejada R.R. Eye-Smoker: A Machine Vision-Based Nose Inference System of Cigarette Smoking Detection using Convolutional Neural Network. Proceedings of the 2020 IEEE 7th International Conference on Engineering Technologies and Applied Sciences (ICETAS).

[43] Zhang D., Jiao C., Wang S. Smoking Image Detection Based on Convolutional Neural Networks. Proceedings of the IEEE 4th International Conference on Computer and Communications (ICCC).

[44] Deng J., Dong W., Socher R., Li L., Li K., Fei-Fei L. ImageNet: A large-scale hierarchical image database. In Proceeding of the IEEE Conference on Computer Vision and Pattern Recognition.

[45] LeCun Y. The MNIST Database of Handwritten Digits. http://yann.lecun.com/exdb/mnist/.

[46] Krizhevsky A. (2009). Learning Multiple Layers of Features from Tiny Images. Master’s Thesis.

[47] Mihalkova L., Moonesy R. Transfer learning from minimal target data by mapping across relational domains. Proceedings of the 21st International Joint Conference on Artificial Intelligence (IJCAI-09).

[48] Szegedy C., Ioffe S., Vanhoucke V., Alemi A. Inception-v4, Inception-ResNet and the Impact of Residual Connections on Learning. Proceedings of the AAAI Conference on Articial Intelligence.

[49] Szegedy C., Vanhoucke V., Ioffe S., Shlens J., Wojna Z. Rethinking the Inception Architecture for Computer Vision. Proceedings of the IEEE Conference on Computer Vision and Pattern Recognition (CVPR).

[50] Chollet F. Xception: Deep Learning with Depthwise Separable Convolutions. Proceedings of the IEEE Conference on Computer Vision and Pattern Recognition (CVPR).

[51] Zoph B., Vasudevan V., Shlens J., Le Q.V. Learning transferable architectures for scalable image recognition. Proceedings of the IEEE Conference on Computer Vision and Pattern Recognition, Salt Lake City.

